# Infections with Soil-Transmitted Helminths in BaAka Pygmies Inhabiting the Rain Forests in the Central African Republic

**DOI:** 10.3390/pathogens13110995

**Published:** 2024-11-14

**Authors:** Wanesa Wilczyńska, Krzysztof Korzeniewski

**Affiliations:** Department of Epidemiology and Tropical Medicine, Military Institute of Medicine—National Research Institute, 04-141 Warsaw, Poland; wanesa.wilczynska@gmail.com

**Keywords:** geo-helminths, deworming, Pygmies, Central African Republic

## Abstract

Poor sanitation, improper food handling, limited access to safe drinking water sources, and limited access to healthcare services contribute to a high prevalence of infections caused by soil-transmitted helminths (STHs) among the BaAka Pygmies, an indigenous community living in Central Africa. The aim of this study was to determine the rates of STH infections in the BaAka people inhabiting the rain forests of the south-western parts of the Central African Republic (CAR) as well as to examine the validity of biannual deworming with a single dose of albendazole 400 mg in high-risk communities exposed to extreme environmental conditions. The study was conducted in August 2021 and involved a sample of 49 BaAka Pygmies inhabiting the rain forest of the Sangha-Mbaéré Prefecture, CAR. The study consisted of collecting single stool samples from each participant and examining the samples for intestinal parasites by light microscopy methods. The collected samples were fixed in SAF fixative and next transported from Africa to Europe, where they were analyzed by light microscopy using three different diagnostic methods (direct smear in Lugol’s solution, the Fülleborn’s flotation, the Kato–Katz thick smear) at the Department of Epidemiology and Tropical Medicine in Gdynia, Poland. Microscopic examination found that 61.2% of the study group were infected with at least one helminthic species. The parasitological screening found invasions with four different species of nematodes, of which hookworm invasions were the most prevalent. The study results demonstrated that although the WHO-recommended mass deworming, which is provided to the BaAka Pygmies in healthcare centers set up on the premises of catholic missions, can effectively reduce the number of infections with soil-transmitted helminths, the prevalence of STH infections remains high in the region. The study findings suggest that in order to contain the spread of STHs in the local community, it will be necessary to implement additional preventive measures, apart from only conducting mass deworming programs.

## 1. Introduction

Intestinal parasitic infections (IPIs) are currently one of the greatest challenges of modern medicine and a significant public health threat that is strongly correlated with poverty and inadequate sanitation. IPIs are primarily found in low-income countries, and they usually affect communities exposed to extreme environmental conditions (including sudden and intense rainfall, cyclones, and high humidity and temperatures). The most prevalent parasitic species responsible for causing gastrointestinal infections in humans include soil-transmitted helminths (STHs), of which *Ascaris lumbricoides*, hookworm (*Ancylostoma duodenale/Necator americanus*), and *Trichuris trichiura* are the most common. Some STH species, e.g., *A. lumbricoides* and *T. trichiura,* are transmitted through the fecal–oral route (by ingestion of food contaminated with the parasite eggs), but hookworm transmission may also occur through direct skin contact with infective larvae [1,2]. The adult stage STHs can survive from 1 up to 10 years in the human host [2]. Transmission of STH infections is facilitated by limited access to safe water sources, poor personal hygiene practices, open defecation practices, walking barefoot, and a lack of health education [3]. It is estimated that approximately 5.3 billion people globally are at risk of acquiring an STH infection, of whom a large majority live in tropical and subtropical regions, mostly in low-income countries in Africa and Asia. Worldwide, approximately 2 billion people are infected with STHs, with the highest prevalence of STH infections being reported from Sub-Saharan Africa [4,5]. STH cases are often asymptomatic or present with mild symptoms. For this reason, they are often left undiagnosed and untreated, and in consequence, they become a source of transmission for other people living in the same community. If left untreated, both symptomatic and asymptomatic STH infections can progress into a chronic illness and lead to protein-energy malnutrition and iron-deficiency anemia (resulting from a mechanical damage of the epithelium), stunted growth in children, nutrient deficiencies, cognitive function disorders, and poor performance [6]. Collectively, STH infections have the highest disability-adjusted life years (DALYs) of all neglected tropical diseases [4]. The Central African Republic is a country with a high birth rate, but very poor water, sanitation, and hygiene conditions. These factors, together with low food security, limited access to safe water sources and limited access to essential healthcare services, contribute to a rapid spread of parasitic diseases among the local people (this especially applies to the diseases transmitted via the oral–fecal route) [7]. Pygmy populations that occupy vast territories of the CAR are an example of a community living under extreme environmental conditions. Pygmy people are indigenous groups of semi-nomadic hunter-gatherers who live in the African rainforests [8]. Because of their nomadic lifestyle, it is difficult to determine the exact size of their population or the geographical distribution of the Pygmy tribes in Africa. However, the data from informal reports released by governmental and non-governmental organizations suggest that there are approximately 100,000 Pygmies living in the Congo Basin alone (mostly in the Democratic Republic of Congo, Congo, and CAR). BaAka people, who form one of the largest Pygmy tribes, occupy the equatorial rainforest west of the Congo river [9]. Traditionally, BaAka Pygmies live from hunting, gathering, fishing, and small-scale farming [10]. As BaAka people have a more diverse diet (including, e.g., wild meat, fish, wild plants, and agricultural plants), they do not suffer such serious malnutrition or nutrient deficits as other populations living in Sub-Saharan Africa [11]. However, they mostly live in places with extremely poor sanitary conditions and are often forced to leave their settlements by the sedentary Bantu people who clear large areas of forests to make space for crops. Pygmy tribes are thought to be inferior by Bantu tribes and have a very low socioeconomic status. BaAka Pygmies have limited access to uncontaminated water sources and very poor knowledge on hygiene practices and health-related risk factors, which all contribute to the spread of parasitic infections, and especially of STH invasions, among their people [12]. The World Health Organization (WHO) recommends regular (annual or biannual) preventive deworming in areas with high prevalence of helminthic infections using a single dose of albendazole (400 mg) or mebendazole (500 mg) [13]. The aim of this study was to determine the rates of STH infections in the BaAka people inhabiting the rainforests in the south-west of the Central African Republic (CAR) and to examine the effectiveness of biannual deworming with a single dose of albendazole 400 mg in high-risk communities which are continuously exposed to extreme environmental conditions. 

## 2. Materials and Methods

### 2.1. Study Population

The screening was conducted in August 2021. The study sample consisted of a group of BaAka Pygmies inhabiting the local rainforests of the Sangha-Mbaéré Prefecture, CAR. CAR is subject to the WHO-recommended deworming program to stop STH infections in Central Africa. The patients were all admitted to a healthcare clinic, which is part of a catholic mission in the village of Monasao. This is a descriptive study with a random selection of participants. Each patient reporting to the clinic could participate in the study, regardless of their age or gender. Information for the patient and informed consent forms were translated into the Sango language which is widely used by the Pygmy people. Due to the widespread illiteracy of the study population, information was provided verbally by medical workers (also Pygmies). The study sample consisted of 49 people aged 17–57 years (the mean age 35.3; females accounted for 39% of the study group), who belonged to a tribal community of about several hundred people (the population of the Pygmy community varied due to their migrations and nomadic lifestyle). 

### 2.2. Sample Collection and Laboratory Procedures

The biological material for parasitological diagnostics consisted of single stool samples (half a 22 mL container), which were self-collected by the study participants and then (on the same day) delivered to the healthcare clinic at the catholic mission in the village of Monasao. After each participant defecated and biological material was collected for testing, they were dewormed with 400 mg of albendazole, which was administered at the same health center. Medical employees of the mission were responsible for the collection of personal data of the study participants and obtaining their informed consent to take part in the procedure. Next, the stool was fixed in SAF fixative (sodium, acetate-acetic, and acid-formalin), and transported to the Department of Epidemiology and Tropical Medicine of the Military Institute of Medicine—National Research Institute in Gdynia, Poland. Parasitological examination of the feces preserved in SAF was performed by light microscopy using three different diagnostic methods (direct smear in Lugol’s solution, the Fülleborn’s flotation, and the Kato–Katz thick smear) [14] a few weeks after collecting biological material for testing. 

### 2.3. Statistical Analysis 

All statistical calculations were performed using Statistica (data analysis software system), version 13 (accessed on 1 August 2024, https://www.statsoft.pl), developed by TIBCO Software Inc. (Palo Alto, CA, USA) (2017). The qualitative variables were presented as counts and percentages. Chi-squared tests for independence were applied to analyze the relationships among categorical variables. A *p*-value of 0.05 was considered the threshold for statistical significance in all analyses. 

### 2.4. Ethical Approval 

Written informed consent was obtained from all the study participants prior to the commencement of the study. The procedure was supervised by Father Wojciech Lula (head of the local catholic mission and of the healthcare clinic in Monasao). The research project was approved by the Committee on Bioethics at the Military Institute of Medicine, Warsaw, Poland (Decision No. 138/WIM/2018, 19 December 2018).

## 3. Results

Of 49 Pygmies participating in the study, 30 were found to be infected with at least one STH species; the prevalence of helminthic infections was found to be 61.2%. The study revealed infections with four different helminthic species: *Ancylostoma duodenale*/*Necator americanus* (hookworm), *Ascaris lumbricoides*, *Trichiuris trichiura*, and *Trichostrongylus* spp., with hookworm being the most prevalent (34.7%). A total of 16.3% of the sample were found to be co-infected with more than one parasitic species, with hookworm and *Trichiuris trichiura* co-infections being predominant (Table 1).

Nearly 70% of women and 60% of men involved in the study were found to be infected with at least one STH species; infections with hookworm were most often found in males, while *Ascaris lumbricoides* infections were most prevalent among females. Polyparasitism was more common in females compared to males (21.2% vs. 13.3%), but has no statistical significance (*p* > 0.05). The distribution of STH infections in the study sample by sex is shown in Table 2. The highest proportion of positive results for STH infections was observed in participants aged between 20 and 30 years old (81.8%), while the lowest percentage of positive results was seen in patients over 40 years old (50.0%) (Table 2). There is an association between hookworm occurrence and age (*p* < 0.05). The greatest number of infections occurred among young adults aged 20–30, probably due to the fact that this group is the strongest and works the hardest. Almost 20% of respondents under the age of 30 were infected with more than one parasite, and the highest rate of co-infestations was observed in the age group 30–40. Laboratory examination was performed by light microscopy using three different diagnostic methods. Direct smear in Lugol’s solution, being the basic screening method, allowed for detection of helminth infections in 26 patients (53.1%). Introduction of thickening methods (the Fülleborn’s flotation and the Kato–Katz thick smear) increased the detection of helminths and the total number of infected patients to 30 persons (62.1%).

Each Pygmy over two years old living in the forest ecosystem was provided with antiparasitic deworming in the form of 400 mg albendazole obtained twice a year at the health center in the catholic mission in Monasao. Deworming performed by medical personnel was irregular due to the nomadic lifestyle of the Pygmies, which involves frequent movements and changes of residence. Nevertheless, in comparison to the studies conducted in 2015 in the same Pygmy community (the same three diagnostic methods were used to detect helminths) [15], it was possible to reduce the rates of STH infections (*p* < 0.0001) (Table 3).

## 4. Discussion

Populations inhabiting the Central African Republic, such as Pygmy communities living in Central African rainforests, are rarely screened or treated for intestinal parasitic infections. There are few publications available in the world’s literature on the morbidity or the prevalence of infectious diseases in this specific ethnic group. Without any doubt, the lifestyle of the Pygmy people, poor sanitation and personal hygiene, as well as limited knowledge on health-related risk factors, all contribute to the spread of many illnesses, while the consumption of unsafe and potentially contaminated food and water is associated with a high risk of IPI transmission [12]. These findings are supported by the results of the study by Korzeniewski et al. [15] which was conducted in 2015. The study demonstrated that as much as 90.5% of the BaAka Pygmies were infected with at least one species of an intestinal parasite, mostly with nematodes (85.0%). The present study, which was conducted 6 years later, on a sample of BaAka Pygmies inhabiting exactly the same region, demonstrated that the prevalence of STH infections was 20% lower than in the previous examination (61.2% vs. 85.02%). The visible reduction in the rates of IPIs in the local community is likely the effect of biannual preventive chemotherapy with a single dose of albendazole (400 mg), which BaAka Pygmies receive at a local healthcare center set up on the premises of a catholic mission in Monasao. The present study found infections by various species of geo-helminths, including infections by four different species of nematodes. Three of the identified nematode species (*Ascaris lumbricoides*, *Trichiuris trichura*, and *Trichostrongylus* spp.) are transmitted through ingestion of food, water, or soil, which is contaminated with the parasite eggs, whereas hookworm infections can be transmitted either through the fecal–oral route or via direct skin contact with soil contaminated with invasive larvae of the parasite (most commonly while walking barefoot on feces-contaminated soil). Because of their lifestyle (e.g., the common practice of walking barefoot), poor personal hygiene practices, improper food handling, and lack of access to safe water sources, it is not surprising that so many BaAka Pygmies participating in the study were found to be infected with hookworm; in fact, hookworm infections were found to be the most prevalent of all STH invasions in the study group [16]. Soil-transmitted helminths are highly endemic in areas inhabited by the BaAka Pygmies. A screening into the prevalence of STH infections in Central African Republic found that as many as 88.2% of the children living in rural areas of the country were infected by helminths, of which *Ascaris lumbricoides* and hookworm invasions were predominant [17]. Parasitological examinations which were conducted in countries neighboring with the Central African Republic found the rates of helminthic infections to be as follows: 58.1–73.8% in the Democratic Republic of the Congo [18,19,20]; 32.5–90.3% in Cameroon [21,22,23]; 2.7–7.8% in Sudan [24,25,26]; and 14–52.1% in Chad [27,28,29,30]. By comparing the above data with the results of the present screening, we have concluded that the BaAka people require continuous support from the international healthcare community. The WHO recommends annual mass drug administration (MDA) in areas where the prevalence of helminths is between 20% and 50% and a biannual MDA in areas where the prevalence of helminths is over 50% [31]. MDA is an effective and inexpensive intervention against STH infections and studies have shown that deworming interventions have a positive impact on the patient well-being, hematological parameters, and nutrition, and in consequence, on their cognitive skills and performance [32]. Due to a high prevalence of STH infections in Central Africa, WHO has implemented a large-scale deworming program in the region. These interventions have been shown to reduce the number of asymptomatic carriers who are an important reservoir for STH transmission [33]. Moreover, preventive chemotherapy with albendazole has been demonstrated to reduce or eliminate other parasitic infections as well, e.g., lymphatic filariasis [34]. Although several studies suggest that MDA, which consists of annual or biannual administration of antiparasitic agents, is effective in reducing the spread of STHs [20,35,36], it is certainly not enough to completely eradicate STH invasions from endemic areas, unless targeted communities change their personal hygiene practices and improve sanitation in their settlements. Apart from administering preventive chemoprophylaxis, it is equally important to educate the populations at risk about the basic preventive measures against STHs, promote wearing footwear, provide them with access to toilets and uncontaminated water sources, as well as with essential healthcare services [15,36]. The results of the present study demonstrate that deworming interventions have been effective in reducing the prevalence of STHs in high-risk communities. It needs to be stressed, however, that MDA alone is not enough to completely eradicate STHs from endemic areas. Also, it is worth pointing out that participation in the deworming program is voluntary, which means that not all members of the local Pygmy community are treated with albendazole, which is yet another obstacle in the fight against helminthiases.

## 5. Conclusions

Poor hygiene and sanitation, lack of access to uncontaminated water sources, poor food handling practices, walking barefoot, and limited access to healthcare services facilitate the transmission of STH infections among the BaAka Pygmies living in the rainforests of the Central African Republic. The WHO recommended mass drug administration, which consists of administering antiparasitic medications at healthcare centers set up on the premises of catholic missions, has been effective in reducing the number of IPIs among the local people; however, the prevalence of parasitic infections among the BaAka people remains high. The BaAka Pygmy population requires continuous support from the international healthcare community, e.g., the provision of appropriate footwear, conducting educational campaigns to increase awareness about the methods to prevent the spread of helminthiases and other infectious diseases, and promoting initiatives to facilitate access to primary healthcare services and to safe water sources. We believe that these interventions, together with MDA campaigns, will reduce the prevalence and transmission of STH infections in the region. 

## Figures and Tables

**Table 1:** Distribution of soil-transmitted helminths detected in the group of BaAka Pygmies (n = 49), Central African Republic, 2021.

Soil-Transmitted Helminths	Number (Percentage) of Infections
Number (percentage) of infected Pygmies	30 (61.2)
Hookworm	17 (34.7)
*Ascaris lumbricoides*	14 (28.6)
*Trichostrongylus* spp.	4 (8.2)
*Trichuris trichiura*	4 (8.2)
Co-infections	8 (16.3)
Hookworm + *Trichostrongylus* spp.	2 (4.1)
Hookworm + *Trichuris trichiura*	3 (6.1)
Hookworm + *Ascaris lumbricoides*	2 (4.1)
*Ascaris lumbricoides* + *Trichuris trichiura* + *Trichostrongylus* spp.	1 (2.0)

**Table 2:** Distribution of soil-transmitted helminths detected in the group of BaAka Pygmies by sex and age (n = 49), Central African Republic, 2021.

Soil-Transmitted Helminths	Number (Percentage) in Pygmies Infected by Sex		*p*-Value	Number (Percentage) of STHs in Pygmies Infected by Age					*p*-Value
	F	M		<20	20–30	30–40	40–50	>50	
Number (percentage) of tested	19 (38.8)	30 (61.2)	0.1161	5 (10.2)	11 (22.4)	11 (22.4)	18 (36.8)	4 (8.2)	0.0116
			0.4106						>0.05
Negative	6 (31.6)	13 (43.3)		2 (40.0)	2 (18.2)	4 (36.4)	9 (50.0)	2 (50.0)	
Positive	13 (68.4)	17 (56.7)		3 (60.0)	9 (81.8)	7 (63.6)	9 (50.0)	2 (50.0)	
Hookworm	6 (31.6)	11 (36.7)	0.7154	1 (20.0) ^a^	8 (72.7) ^a,b,c^	4 (36.4)	4 (22.2) ^b^	0 (0.0) ^c^	<0.05 ^a,b,c^
*Ascaris lumbricoides*	7 (36.8)	7 (23.3)	0.3078	2 (40.0)	2 (18.2)	4 (36.4)	5 (27.8)	1 (25.0)	>0.05
*Trichostrongylus* spp.	3 (15.8)	1 (3.3)	0.1208	0 (0.0)	0 (0.0)	2 (18.2)	1 (5.6)	1 (25.0)	>0.05
*Trichuris trichiura*	2 (10.5)	2 (6.7)	0.6307	1 (20.0)	1 (9.1)	2 (18.2)	0 (0.0)	0 (0.0)	>0.05

^a^ Hookworm is significantly more common in the group of 20–30-year-olds than in the group < 20. ^b^ Hookworm is significantly more common in the group of 20–30-year-olds than in the group 40–50. ^c^ Hookworm is significantly more common in the group of 20–30-year-olds than in the group > 50.

**Table 3:** Distribution of soil-transmitted helminths detected in the group of BaAka Pygmies in 2015 (n = 950) and in 2021 (n = 49), Central African Republic, 2021.

Soil-Transmitted Helminths	Number (Percentage) of STHs in Pygmies Tested in 2015	Number (Percentage) of STHs in Pygmies Tested in 2021	*p*-Value
Total number (percentage) of infections	1827 (85.0)	30 (62.1)	<0.0001
Hookworm	633 (66.6)	17 (34.7)	<0.0001
*Ascaris lumbricoides*	640 (67.3)	14 (28.6)	<0.0001
*Trichuris trichiura*	229 (24.1)	4 (8.2)	0.0101
*Trichostrongylus* spp.	169 (17.8)	4 (8.2)	0.0825
*Strongyloides stercoralis*	114 (12.0)	0 (0.0)	0.0100
*Capillaria* spp.	35 (3.7)	0 (0.0)	0.1714
*Enterobius vermicularis*	7 (0.7)	0 (0.0)	0.5465
*Hymenolepis nana*	3 (0.3)	0 (0.0)	0.6936
*Taenia* spp.	2 (0.2)	0 (0.0)	0.7478

## Data Availability

The data presented in this study are available on request from the corresponding author.
